# A227 THE EFFECTIVENESS OF PEG 3350 COMPARED TO LACTULOSE FOR THE TREATMENT OF ACUTE HEPATIC ENCEPHALOPATHY IN ADULT CIRRHOTIC PATIENTS: A SYSTEMATIC REVIEW AND META-ANALYSIS

**DOI:** 10.1093/jcag/gwab049.226

**Published:** 2022-02-21

**Authors:** T Afzaal, C J Karvellas, J Dionne

**Affiliations:** 1 Department of Medicine, McMaster University, Hamilton, ON, Canada; 2 Hepatology/Critical Care Medicine, University of Alberta, Edmonton, AB, Canada

## Abstract

**Background:**

Cirrhosis is the leading cause of liver-related death globally. Hepatic encephalopathy (HE) leads to significant morbidity and mortality. Lactulose is the current gold standard treatment for HE; it eliminates nitrogenous waste from the gut. Polyethylene glycol 3350–electrolyte solution (PEG) is a safe, common and effective purgative with recent studies suggesting its efficacy resulting in faster resolution of HE and shorter hospital length of stay.

**Aims:**

To assess the efficacy and safety of PEG 3350 compared to lactulose in adult cirrhotic patients 18 years of age and older with overt hepatic encephalopathy on patient important outcomes including: improvement of hepatic encephalopathy, hospital length of stay and mortality.

**Methods:**

We reviewed databases MEDLINE, EMBASE, OVID, CINAHL, Cochrane Database, PubMed, Trip database, the grey literature, and clinicaltrials.gov from inception to December 2020: PROSPERO CRD42021257641. Search strategy was developed in conjunction with medical librarian. Randomized controlled trials (RCTs), either published or non-published, were included in the review. Continuous data was analyzed using mean difference with random-effects model. Dichotomous data was analyzed using the Mantel-Haenszel method using random-effects model. Statistical effect-size heterogeneity was assessed using Chi^2^ test and quantifying the relative proportion of variation using I^2^ statistic. The overall certainty of evidence will be assessed using the Grading of Recommendations, Assessment, Development and Evaluations system (GRADE).

**Results:**

From the 68 studies, 16 were assessed for full text review from which 5 studies were included in the meta-analysis representing a total of 351 patients. The primary outcome of mean change in Hepatic Encephalopathy Scoring Algorithm (HESA) at 24-hours from baseline demonstrated an improvement in the PEG group compared to lactulose group [Mean difference (MD)= 0.60, 95% CI (0.20, 1.01)]. In comparison to lactulose, PEG also demonstrated a shorter hospital length of stay [MD = -1.00, 95% CI (-1.99, -0.01)], shorter time to HE resolution [MD= -1.49, 95% CI (-1.81, -1.16)] and showed a mortality benefit [RR=0.35, 95% CI (0.13 to 0.92)]. There was no significant difference between change in ammonia levels at 24 hours [MD= -25.80, 95% CI (-95.39, 43.78)].

**Conclusions:**

PEG leads to a faster improvement and resolution of HE when compared to the current standard of care, lactulose.

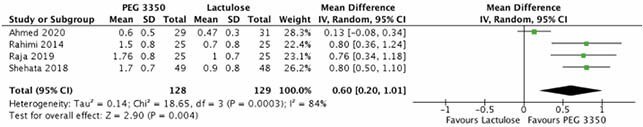

**Funding Agencies:**

None

